# Heat-Treated Lysozyme Hydrochloride: A Study on Its Structural Modifications and Anti-SARS-CoV-2 Activity

**DOI:** 10.3390/molecules28062848

**Published:** 2023-03-21

**Authors:** Serena Delbue, Elena Pariani, Silvia Parapini, Cristina Galli, Nicoletta Basilico, Sarah D’Alessandro, Sara Pellegrino, Elena Pini, Samuele Ciceri, Patrizia Ferraboschi, Paride Grisenti

**Affiliations:** 1 Department of Biomedical, Surgical and Dental Sciences, Università degli Studi di Milano, 20133 Milan, Italy; 2 Department of Biomedical Sciences for Health, Università degli Studi di Milano, 20133 Milan, Italy; 3 Department of Pharmacological and Biomedical Sciences, Università degli Studi di Milano, 20133 Milan, Italy; 4 Department of Pharmaceutical Sciences, General and Organic Chemistry Section “Alessandro Marchesini”, University of Milan, 20133 Milan, Italy; 5 Department of Pharmaceutical Sciences, University of Milan, Via L. Mangiagalli 25, 20133 Milan, Italy; 6 Department of Medical Biotechnology and Translational Medicine, Università degli Studi di Milano, Via Saldini 50, 20133 Milan, Italy; 7 Bioseutica, Landbouwweg 83, 3899 BD Zeewolde, The Netherlands; 8 Bioseutica, Corso Elvezia 4, 6900 Lugano, Switzerland

**Keywords:** peptidoglycan-degrading enzyme, innate immunity, antibiotic activity, antiviral activity, NMR, FTIR

## Abstract

Lysozyme (E.C. 3.2.1.17), an about 14 kDa protein and pI 11, widely spread in nature, is present in humans mainly in milk, saliva, and intestinal mucus as a part of innate defense mechanisms. It is endowed with antimicrobial activity due to its action as an N-acetylmuramidase, cleaving the 1-4β glycosidic linkage in the peptidoglycan layer of Gram-positive bacteria. This antimicrobial activity is exerted only against a limited number of Gram-negative bacteria. Different action mechanisms are proposed to explain its activity against Gram-negative bacteria, viruses, and fungi. The antiviral activity prompted the study of a possible application of lysozyme in the treatment of SARS-CoV-2 infections. Among the different sources of lysozyme, the chicken egg albumen was chosen, being the richest source of this protein (c-type lysozyme, 129 amino acids). Interestingly, the activity of lysozyme hydrochloride against SARS-CoV-2 was related to the heating (to about 100 °C) of this molecule. A chemical–physical characterization was required to investigate the possible modifications of native lysozyme hydrochloride by heat treatment. The FTIR analysis of the two preparations of lysozyme hydrochloride showed appreciable differences in the secondary structure of the two protein chains. HPLC and NMR analyses, as well as the enzymatic activity determination, did not show significant modifications.

## 1. Introduction

Lysozyme (also named Muramidase) is a hydrolytic enzyme endowed with antibiotic and antiviral properties, first discovered by A. Fleming [1]. Lysozyme is widely found in nature and detected not only in hen egg white (HEW) but also found in tears, nasal mucus, milk, saliva, and blood serum; in a great number of other tissues and secretions of different animals, including both vertebrates and invertebrates; in some molds, and in the latex of different plants [2].

Due to their different origins, different lysozyme types were identified, all having the common feature of cleaving the β-(1,4)-glycosidic bonds between N-acetylmuramic acid and N-acetylglucosamine in peptidoglycan, the major bacterial cell wall polymer, thereby causing bacterial cell lysis via the targeting of bacterial cell walls, which are critical for the resistance of bacteria to osmotic stress [3]. On the basis of their amino acid sequence and biochemical properties, three types of lysozymes have been described, labelled as c-type (chicken type), g-type (goose type) and i-type (invertebrate type). In addition to their enzymatic activity, c-type lysozymes have a high ratio of hydrophobic and positively charged amino acid residues, and can insert into and form pores in negatively charged bacterial membranes [4]. Both the enzymatic and cationic features of c-type lysozymes have been implicated in antibacterial activity.

This molecule, endowed with a broad spectrum of antiviral and antibacterial action [2], is also able to stimulate the immune response; its lytic properties against some viruses and bacteria can release molecules with immune activity, increase serum and tissue glutathione levels, and influence increased lymphocyte proliferation. The antiviral activity of lysozyme was confirmed against several viruses, including: Herpes virus (Simplex and Zoster); Norovirus; Poliovirus; Epstein–Barr virus; and Varicella-Zoster virus [5,6,7,8].

Cisani et al. [8] have described lysozyme antiviral activity against herpes virus only when heat treated (i.e., treated at 100 °C for 15′), without exploring the structural changes to lysozyme after this thermal treatment. This heat treatment in aqueous solution is supposed to completely inactivate the enzymatic activity of lysozyme, but without reports of any supporting data. Other authors obtained similar results testing a heat-denatured lysozyme in aqueous solution against murine Norovirus [5]. These studies suggested that the enzymatic and antiviral activity of native and heat-treated lysozyme are not necessarily associated, and that the employed experimental conditions affected the native structure of lysozyme with the simultaneous formation of a complex mixture of degradation products and of oligomeric forms in which dimer, trimer, and tetramer forms can be identified. However, from the above-cited literature data, it is not clear if the antiviral activity of lysozyme is due to its residual enzymatic activity, to its ionic properties, or to the presence of degradation products, such as aggregates or oligopeptides.

On the basis of heat treatment tests of lysozyme under different experimental conditions (in aqueous solution and on powder) we were aware that the heating of solid lysozyme HCl at 100 °C for 40′ does not denature this protein, since it presents the same enzymatic activity as the native hen egg white lysozyme, HEWL (enzymatic activity determined by the *Micrococcus luteus* test), the same structural integrity of native HEWL (determined by mass and NMR spectroscopy), and the absence of degradation products and aggregates (determined by HPLC and SDS PAGE). After this controlled heat treatment, carried out on the solid lysozyme HCl, only FTIR spectroscopy, circular dichroism, and far-UV circular dichroism give us the evidence of a change in the secondary structure of this protein, with an increase of the percentage of helical structure when compared to native HEWL. This structural change is stable over time and also detectable in aqueous solution.

Recently, the potential therapeutic use of lysozyme as antiviral and immune-modulating agent against COVID-19 was proposed [9]. In fact, the antimicrobial effects of this molecule against a wide range of microorganisms could suggest its use upon presentation of COVID-19 symptoms, in order to prevent noncritical cases from progressing to critical cases. For instance, this compound might act against bacteria and fungi responsible for secondary infections in COVID-19 patients [10]; lysozyme could have beneficial effects in counteracting pathological features occurring in severe COVID-19 infections (e.g., neutrophil infiltration, macrophage activation, free iron overload, oxidative stress, advanced glycation end-product (AGE) effects, excessive proinflammatory cytokine production, and thrombus formation); and its immunomodulatory properties might promote the resolution of inflammation. Moreover, lysozyme, when subjected to simulated gastrointestinal digestion, afforded a hydrolysate endowed with angiotensin I-converting enzyme (ACE) inhibitory (IC_50_ = 12.6 μg/mL) and marked antioxidant activity [11].

More recently, the results of specific tests carried out on Caco-2 cells have suggested that native lysozyme (purified from human neutrophils and from hen egg whites) does not protect against SARS-CoV-2 infection, as it does not show direct antiviral activity [12].

Taking into account that the literature data suggest antiviral activity mainly associated with heat-treated lysozyme, we decided to study the antiviral activity of native (i.e., not heat-treated) and heat-treated lysozyme hydrochloride against SARS-CoV-2, and to better define the structure of lysozyme hydrochloride after heat treatment.

## 2. Results and Discussion

### 2.1. Compound Cytotoxicity

**Figure 1:**
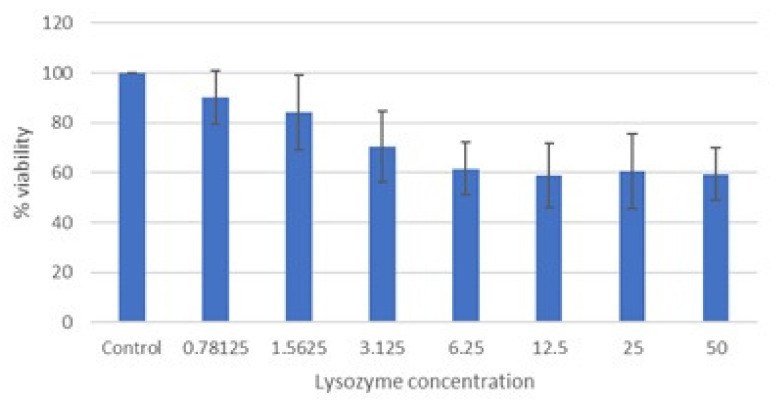
Cytotoxicity of lysozyme HCl (mg/mL) after 72 h of incubation (MTT assay). Cell viability expressed as the percentage of control (untreated) cell viability. Data are expressed as the means ± SD of three independent experiments performed in duplicate.

The cytotoxicity of lysozyme HCl was measured by MTT assay (3-(4,5-dimethylthiazol-2-yl)-2,5-diphenyltetrazolium bromide) on Vero cells. Concentrations of lysozyme HCl between 50 and 6.25 mg/mL inhibited cell viability by more than 30% (Figure 1).

The CC_50_ and CC_10_ of lysozyme HCl are 13.3 ± 1.7 and 1.19 ± 0.14, respectively. The cytotoxicity data of lysozyme HCl are consistent with the literature data [13].

Based on these data, the maximum nontoxic concentration of lysozyme HCl was assumed to be 1 mg/mL; therefore, this concentration and subsequent concentrations were selected in order to test antiviral activity.

### 2.2. Antiviral Activity of Lysozyme HCl against SARS-CoV-2

**Figure 2:**
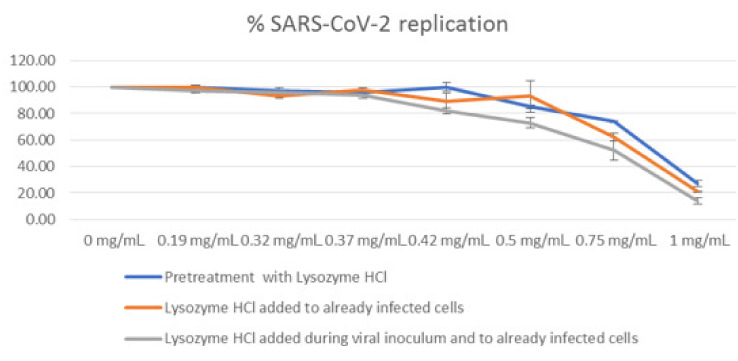
Antiviral activity of lysozyme HCl against SARS-CoV-2, expressed as percentage of viral replication.

Table 1 and Figure 2 summarize the results obtained by qRT-PCR, after heating the cell supernatants at 98 °C for 5 min. Results are expressed as the percentage of SARS-CoV-2 replication (mean of at least three experiments).

The highest activity was obtained when lysozyme HCl was pre-incubated with the virus and re-added to already-infected cells (pretreatment + post treatment). Under this condition, lysozyme HCl at the concentrations of 0.50 mg/mL, 0.75 mg/mL and 1.00 mg/mL causes a decrease of 27%, 48%, and 86% in viral replication, respectively. The lysozyme HCl antiviral activity was lower when the compound was added only to already-infected cells (post treatment), and even lower when it was only pre-incubated with the virus (pretreatment).

### 2.3. Lysozyme Characterization

Considering the biological results described above, we investigated the changes that occur on lysozyme HCl before and after heat treatment by different analytical techniques.

The NMR (^1^H-^13^C HSQC, ^1^H-^15^N HSQC and solid state ^13^C-NMR) and mass spectroscopy analyses of lysozyme HCl before and after heat treatment were practically superimposable, and the absence of degradation products was verified by HPLC and SDS-PAGE analyses. Particularly, the presence of aggregates deserves a comment: the presence of dimer, trimer, and tetramer forms after heating lysozyme is well known [14], even if the experimental conditions to generate aggregates are not fully reported, and this might create misleading data. We decided to utilize lysozyme HCl, instead of lysozyme base, in our trials since. As shown by our analyses, the hydrochloride salt is stable to heat degradation, under the experimental conditions (time and temperature) followed by us, especially working on the powder. The presence of potential degradation products and aggregates on lysozyme can be detected by HPLC and SDS-PAGE. Indeed, for example, the presence of lysozyme as a dimeric form could be detected on the HPLC chromatogram at a relative retention time of about 1.1 and on the SDS-PAGE utilizing a marker proteins mixture in the molecular weight range of about 10–60 kDalton.

Moreover, the *Micrococcus luteus* test confirmed that significant changes in the enzymatic activity did not occur.

Fourier transform infrared (FTIR) spectroscopy is widely used in the structural characterization of proteins or peptides. This study, applied to the amide I region between 1700 and 1600 cm^−1^ of the spectrum, is utilized in order to evaluate the secondary structure of proteins, as it enables the resolution of the amide peak into smaller component peaks, each representative of a different secondary structure [15,16]. In this region falls the larger contribution of C=O stretching vibrations of the amide group on the protein backbone. The peaks between 1650–1658 cm^−1^ are assigned to alpha helix; 1620–1640 cm^−1^ and 1670–1695 cm^−1^ to beta sheet; 1640–1648 cm^−1^ to unordered conformation; and around 1670, 1683, 1688, and 1694 to beta-turns.

Some mathematical techniques, such as deconvolution and second derivative, are necessary to visualize the overlapping bands. In the second derivative, a minimum corresponds to a local maximum in the original spectrum recorded in absorbance mode. However, derivatives are especially sensitive to noise in the original spectrum [17] and the peak positions are sometimes difficult to distinguish.

The deconvolution process, in addition to showing a better distinction of the overlapping CO-NH peaks, allows the estimation of percentages associated with each secondary structure component.

**Figure 3:**
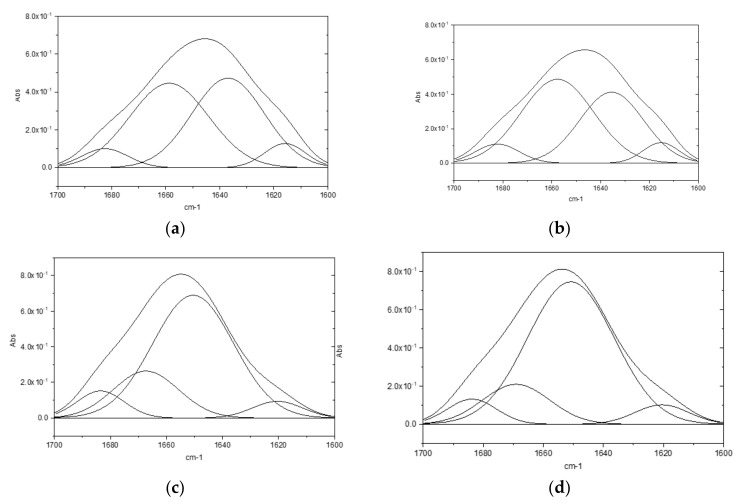
ATR-FTIR deconvolution: (**a**) lysozyme HCl powder; (**b**) heat-treated lysozyme HCl powder; (**c**) lysozyme HCl aqueous solution (2 mg/mL); (**d**) heat-treated lysozyme HCl aqueous solutions (2 mg/mL).

Results of the ATR-FTIR deconvolutions performed on lysozyme powder highlight no significant differences in secondary structure conformation following heat treatment (Figure 3a,b) However, differences between untreated and heat-treated lysozyme were observed in the aqueous solutions (Figure 3c,d).

In Table 2, chemical shifts, secondary structure attribution [18], and corresponding percentage are reported. Due to a different protein folding in aqueous medium, the percentage of α helix in the powders is lower than in the solutions and a turn structure appears. Moreover, compared to native lysozyme, the α helix structure increases in the heat-treated one. This is possibly correlated to the enhanced activity of said protein, as it is established in the literature [19] that the protein’s stability is related to an increase of the α helix percentage in its structure.

**Figure 4:**
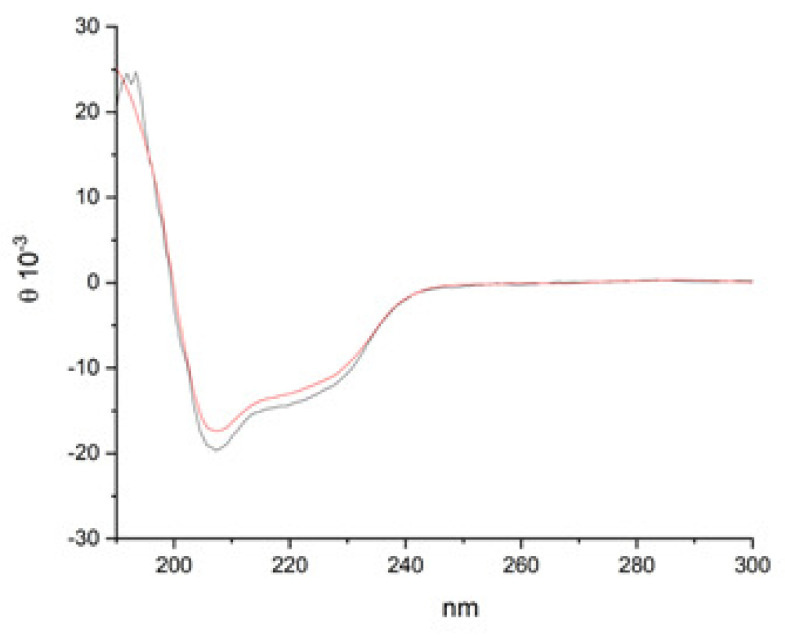
CD spectra of lysozyme HCl (black line) and heat-treated lysozyme HCl (red line) in water (0.25 mg/mL).

In order to further characterize the two proteins, far-UV, and circular dichroism (CD) analyses were performed on lysozyme HCl and heat-treated lysozyme HCl at a final concentration of 0.25 mg/mL in water (Figure 4). In the amide bond region, two negative minima at 225 and 208 nm, and a positive band at 200 nm were observed. In particular, the presence of the exciton splitting of the π → π* transition band indicates a preferred helix conformation. These results are in accordance with the literature data on lysozyme [20,21,22]. On the other hand, the R = [θ]∼222 nm/[θ]∼208 nm values are around 0.7 for both proteins, suggesting the presence of both alpha and 3_10_ helical structures [23].

Increasing the protein concentration to 0.5 mg/mL (Supplementary Materials Figure S13), the two proteins both maintain an overall helical conformation, although the scattering of the signals at 190 nm and the increase of intensity at 208 nm in the spectrum of lysozyme HCl could be ascribed to the presence of nonhomogeneous aggregates.

## 3. Materials and Methods

### 3.1. Chemical–Physical Characterization of Lysozyme HCl

Native lysozyme HCl and heat-treated lysozyme HCl (LysOHT^®^) were supplied by BIOSEUTICA BV (Landbouwweg 83, 3899 BD Zeewolde, The Netherlands).

Heat-treated lysozyme HCl was prepared from native lysozyme HCl by heating the same at 90–100 °C in an oven for 40′ and the obtained product was stored at room temperature.

#### 3.1.1. Enzymatic Activity

The enzymatic activity of native lysozyme HCl, as well as that of heat-treated lysozyme HCl, was determined using the *Micrococcus luteus* test (FIP method test according to Ref. [24]) showing a value of 41,726 and 41,472 FIP unit/mg, respectively. This difference in the enzymatic activity was negligible.

#### 3.1.2. HPLC Analyses

The chromatographic profile of utilized native lysozyme, as well as that of heat-treated lysozyme HCl, was determined using a chromatographic HPLC method employing the following analytical conditions: HPLC column TSK-GEL Phenyl-5PW RP 75 × 4.6 mm ID, 10 µm Tosoh; wavelength 281 nm; column temperature 30 °C; flow rate: 1 mL/min; eluant A: acetonitrile/water 10: 90 *v*/*v* + trifluoroacetic acid (TFA) 0.2% *v*/*v*; eluant B: acetonitrile/water 70: 30 *v*/*v* + TFA 0.2% *v*/*v*; gradient elution according to the composition indicated in Table 3, reported below.

**Figure 5:**
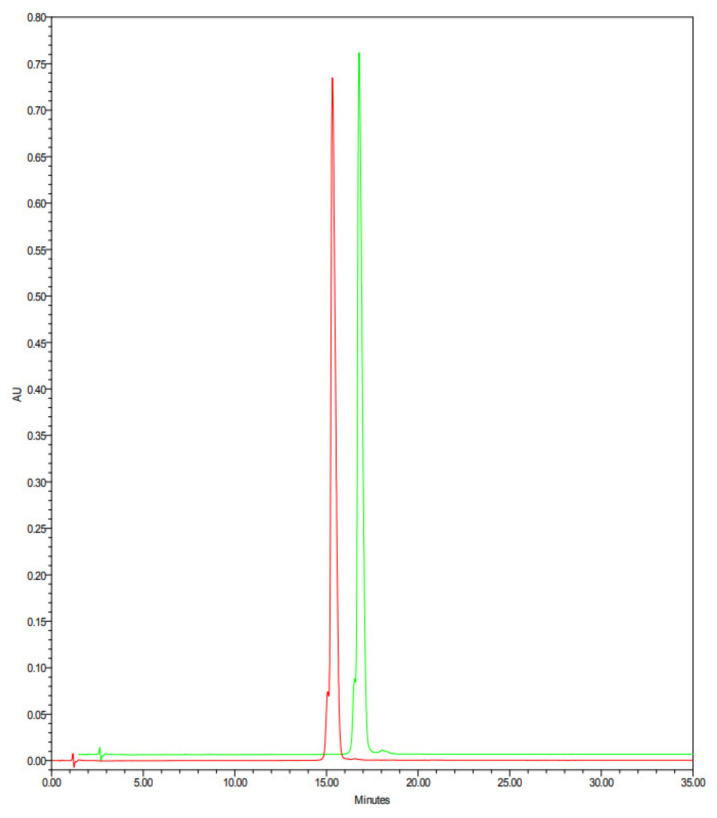
HPLC chromatograms of native lysozyme HCl (red line) and heat-treated lysozyme HCl (green line).

Representative HPLC chromatograms of native lysozyme HCl (red line) and heat-treated lysozyme HCl (green line), analyzed with the above reported analytical method, are reported in Figure 5. These HPLC analyses are practically superimposable, and no evidence of new degradation products are present on heat-treated lysozyme HCl.

#### 3.1.3. SDS-PAGE

SDS-PAGE analyses (reported in the Supplementary Materials) were performed using an Invitrogen Mini Gel Tank instrument and a Novex Wedge gel 4 to 20% Tris-Glycine, 1.0 mm, Mini Protein Gel 10Well by seeding the following volumes of sample solutions (from left to right):

(1) LMW calibration kit for SDS electrophoresis (from 14.4 to 97.0 Dalton): 5 µL
(2) Lysozyme HCl: 8 µg
(3) LysOHT^®^: 8 µg
(4) Lysozyme HCl heat treated in aqueous solution (0.1% aqueous solution for 20′ at 100 °C): 8 µg

The seeding solution of lysozyme HCl and LysOHT^®^ was prepared at the concentration of 1 mg/mL in water/tris-glycine SDS sample buffer for wells 2–3. Well number 4 was seeded at the concentration of 0.5 mg/mL. These solutions, before the seeding, were heated for 5–10 min at 85 °C in a block heater. The gel was developed with Imperial Stain (Thermo Scientific, Waltham, MA, USA) for 1 h under gentle stirring.

#### 3.1.4. HPLC/HRMS Analyses

HPLC/HRMS analyses were performed with an HPLC Platinblue system (Knauer, Berlin, Germany) coupled with an Impact II mass spectrometer (Bruker, Billerica, MA, USA). HPLC column: Luna C8 (2) 100 Å 150 × 2.0 mm ID, 3 µm; wavelength 280 nm; column temperature 45 °C; flow rate: 0.1 mL/min; sample concentration: 1.0 mg/mL in water; injection volume: 1 μL; eluant A: 0.1% *v*/*v* aqueous solution of formic acid; eluant B: acetonitrile (HPLC grade); gradient elution according to the composition is indicated in Table 4, reported below.

The mass spectrometer was set up as follows: Capillary voltage: −4500 V; nebulizer needle: 1.8 bar; dry gas flow rate: 8.0 L/min; dry gas temperature: 220 °C; mass range: 300–3000 *m*/*z*.

The mass spectra are available in the Supplementary Materials section.

#### 3.1.5. NMR Spectroscopy

^1^H-^13^C HSQC and ^1^H-^15^N HSQC NMR spectra were recorded on a Bruker Avance III 600 MHz spectrometer (Bruker, Billerica, MA, USA), equipped with a 5 mm TCI cryogenic probe operating at 600.13, 150.90, and 60.83 MHz for ^1^H, ^13^C, and ^15^N, respectively. The NMR experiments were performed by dissolving the samples in D_2_O (isotopic enrichment 99.9 atom % D) or H_2_O according to the following acquisition parameters:

*Spectra recorded from sample solutions (100 mg/mL in D_2_O) at 303 K*.

- ^1^H NMR: 16 scans, delay between scans = 6 s
- ^1^H-^13^C HSQC: 16 scans, delay between scans = 2.5 s, number of increments = 640, ^1^H spectral range = from −3 to 12 ppm, ^13^C spectral range = from −5 to 155 ppm
- ^13^C NMR: 8192 scans, delay between scans = 2.5 s

*Spectra recorded from sample solutions (100 mg/mL in H_2_O/D_2_O 9:1 v/v) at 303 K.*

- ^1^H NMR: 16 scans, delay between scans = 6 s
- ^1^H-^15^N HSQC: 48 scans, delay between scans = 2.0 s, number of increments = 512, ^1^H spectral range = from 5.5 to 12.5 ppm, ^15^N spectral range = from 95 to 135 ppm

Solid-state NMR spectra were recorded on a Bruker AV500HD spectrometer (Bruker, Billerica, MA, USA), equipped with a 3.2 mm MAS double-resonance probe operating at 500.13 and 125.76 for ^1^H and ^13^C, respectively. For cross-polarization, a ramped rf field was applied on the ^1^H channel. The cross-polarization contact time was 51 ms. A recycle delay of 7.8 s was used. The ^1^H and ^13^C 90° pulse lengths were 2.27 and 3.57 ms, respectively. ^13^C chemical shifts are referenced by using the ^13^C resonance of solid glycine at 43.31 ppm as an external reference.

The NMR spectra are available in the Supplementary Materials section.

#### 3.1.6. FTIR Analyses

The FTIR experiments were performed on a Perkin Elmer Spectrum One FTIR (Perkin Elmer, Waltham, MA, USA), equipped with a Perkin Elmer Universal ATR sampling accessory consisting of a diamond crystal. Spectra were obtained from 4000 to 650 cm^−1^, with a resolution of 4 cm^−1^, for a total of 32 scans, and each analysis was repeated three times. CO_2_ and water vapor spectra were subtracted to eliminate possible environmental interferences. In the solid-state analysis, the samples were placed on the diamond, ensuring complete coverage of its surface, and pressure was applied with the specific punch. The resulting spectra were elaborated utilizing the ATR correction algorithm on the instrument. Using the FTIR instrument second derivative, the deconvolution of the amide I region (1700–1600 cm^−1^) was performed utilizing the Origin Pro software. Protein films, prepared by slow water evaporation on the ATR plate of 10 µL of 2 mg/mL distilled water solution, were also analyzed in the same conditions.

#### 3.1.7. Circular Dichroism (CD) Analyses

The CD experiments were performed on a Jasco 810 CD-spectropolarimeter. Spectra were obtained from 190 to 300 nm with a 0.1 nm step and 1 s collection time per step, taking three averages. The spectrum of the solvent was subtracted to eliminate interference from cell, solvent, and optical equipment. The CD spectra were plotted as mean residue ellipticity θ (degree × cm^2^ × dmol^−1^) versus wavelength λ (nm). Noise reduction was obtained using a Fourier transform filter program from Jasco. Spectra were recorded at a final protein concentration of 0.25 mg/mL and 0.5 mg/mL in water.

#### 3.1.8. Zeta Potential Analyses

Zeta potentials were measured using the Zetasizer Nano ZS (Malvern, Worcestershire, UK) with a fixed scattering angle of 173° and a 633 nm helium–neon laser. Data were analyzed using Zetasizer software version 7.11 (Malvern, Worcestershire, UK). Disposable plastic micro cuvette DTS1070 cuvettes for zeta potential analysis were used.

The zeta potential determinations are available in the Supplementary Materials section.

### 3.2. Cells and Virus

Vero cells (CCL-81, Monkey Kidney Epithelial Cells) were purchased at ATCC (Virginia, USA). Cells were maintained in DMEM medium (Dulbecco’s Modified Eagle’s Medium) supplemented with 10% heat inactivated fetal calf serum, 2 mM glutamine, 100 units/mL of penicillin, 100 μg/mL of streptomycin.

SARS-CoV-2 was isolated from the nasopharyngeal swab of a COVID-19 patient, upon approval from the Local Ethics Committee and a signature of informed consent (The Fondazione Ca’ Granda, Ospedale Maggiore, Milano, Italy, approved the protocol No. 456_2020 in May 2020). The isolated SARS-CoV-2 strain belongs to the B.1 lineage, carrying the characteristic spike mutation D614G. The B.1 lineage is the large European lineage, the origin of which roughly corresponds to the Northern Italian outbreak in early 2020. The complete nucleotide sequence has been deposited at GenBank and GISAID (accession Nos. MT748758.1 and EPI_ISL 584051, respectively).

### 3.3. Cytotoxicity Assay

Cell toxicity was monitored by determining the effect of lysozyme HCl on Vero cells. For the cytotoxicity assay, cells were seeded into 96-well plates at a concentration of 1 × 10^4^ cells/well. After 24 h of incubation, the cells were treated with serial 2-fold dilutions of lysozyme HCl (from 50 to 0.05 mg/mL), in a final volume of 200 μL, in duplicate. After incubation for 72 h at 37 °C in 5% CO_2_, cell viability was measured by MTT assay [25]. The percentage of viable cells was calculated, using untreated cells as control (100% viability), utilizing the formula:

[(sample absorbance—cell free sample blank)/mean media control absorbance] × 100

The 50% cytotoxic concentration (CC_50_) causing 50% reduction of Vero cell viability with respect to untreated control cells was determined using Gen5 software. The morphological changes of Vero cells were also observed by light microscopy.

### 3.4. Vero Cells Infection and Compounds Treatment

Vero cells were seeded into 96-well plates at a density of 1.3 × 10^4^ cells/well and were incubated for 24 h at 37 °C, 5% CO_2_. Infection and treatment were performed following three different protocols:

- Pretreatment of virus particles: SARS-CoV-2 (multiplicity of infection, MOI, of 0.05) was first incubated for 1 h at 37 °C in the presence of different concentrations of compounds, and then added to the cell monolayer, for 2 h at 37 °C, 5% CO_2_.
- Post treatment: cells were infected with SARS-CoV-2 (MOI of 0.05); after removal of virus inoculum, the cells were treated with medium containing different concentration of compounds.
- Pretreatment + post treatment: SARS-CoV-2 (MOI of 0.05) was first incubated for 1 h at 37 °C in the presence of different concentrations of compounds, and then added to the cell monolayer and incubated for 2 h at 37 °C, 5% CO_2_; after removal of virus inoculum, the cells were re-treated with medium containing a different concentration of compounds.

In all the three protocols, the cells were maintained for 72 h at 37 °C, 5% CO_2_, after infection and treatment.

Three independent experiments were performed in triplicate.

### 3.5. Evaluation of the Antiviral of the Compounds by Quantitative Real Time RT-PCR

Quantification of SARS-CoV-2 copy numbers in cell supernatants was evaluated via specific real time RT-PCR, (qRT-PCR) of the N1 gene, after heat treatment of the supernatant at 98 °C for 5 min, according to the protocols [26,27]. Results were expressed as the average +/− Standard Deviation (SD) of the percentage of viral replication, considering the replication in infected untreated Vero cells as 100%.

## 4. Conclusions

The antiviral activity of lysozyme HCl against SARS-CoV-2 seems to be strictly related to the heat treatment of this compound. This activity, absent in native lysozyme HCl, appears only after heating of the cell supernatants containing lysozyme HCl at 98 °C and after direct heating of lysozyme HCl at 90–100 °C for 40′, and was found to be dose dependent.

The modifications in the structure of lysozyme HCl after heat treatment were investigated by HPLC/HRMS, ^1^H-^13^C HSQC, ^1^H-^15^N HSQC, solid-state ^13^C-NMR, zeta potential measurement, LC-MS, SDS-PAGE, and HPLC analyses. These analyses confirmed that the applied heating conditions did not modify the spectroscopic properties of the heat-treated lysozyme HCl, when compared to the native lysozyme HCl, and did not lead to the formation of degradation products or aggregates (dimers, trimers, and tetramers). Additionally, the enzymatic activity, determined through the *Micrococcus luteus* test, and the HPLC impurity profile of the heat-treated lysozyme HCl were unchanged compared to those of native lysozyme HCl. FTIR spectroscopy, circular dichroism, and far-UV circular dichroism performed on the solid-state lysozymes highlight no significant differences in secondary structure conformation following the heat treatment; however, significant differences have been observed in the aqueous films. The results of these analyses highlight a higher percentage of helical structure in the heat-treated lysozyme, possibly correlated with the antiviral activity of said protein.

## 5. Patents

The results of this work are subject to International Patent application number WO20217229430 A1 (filing date 11/05/2021)

## Figures and Tables

**Table 1:** Antiviral activity of lysozyme HCl against SARS-CoV-2, expressed as percentage of viral replication ± SD.

Concentration of Lysozyme HCl (mg/mL)	Percentage of SARS-CoV-2 Replication ± SD		
	Pretreatment	Post Treatment	Pretreatment + Post Treatment
0.00	100	100	100
0.19	100 ± 1.50	100 ± 1.22	97 ± 1.52
0.32	97 ± 2.71	93 ± 1.26	96 ± 3.23
0.37	96 ± 2.25	98 ± 1.25	94 ± 2.19
0.42	100 ± 3.19	89 ± 6.45	82 ± 2.37
0.50	n/a	93 ± 11.97	73 ± 3.83
0.75	74 ± 0.69	62 ± 3.01	52 ± 7.02
1.00	27 ± 2.30	21 ± 0.78	14 ± 2.05

**Table 2:** Deconvolution results of ATR-FTIR analyses.

Sample	α-Helix cm^−1^ (%)	β-Sheet cm^−1^ (%)	Turns cm^−1^ (%)
Lysozyme HCl powder	1659 (44%)	1615 (7%); 1636 (43%); 1683 (6%)	-
Heat-treated lysozyme powder	1657 (50%)	1615 (6%); 1635 (37%); 1682 (7%)	-
Lysozyme HCl aq. solution	1650 (66%)	1620 (6%); 1683 (8%);	1667 (20%)
Heat-treated lysozyme aq. solution	1651 (72%)	1620 (6%); 1684 (7%)	1669 (15%)

**Table 3:** HPLC gradient elution for lysozyme HCl analyses.

**Time (Minutes)**	Eluant A (% *v*/*v*)	Eluant B (% *v*/*v*)
0.0	100.0	0.0
35.0	0.0	100.0
35.1	100.0	0.0
42.0	100.0	0.0

**Table 4:** Gradient elution for HPLC/HRMS analyses of lysozyme HCl.

Time (Minutes)	Eluant A (% *v*/*v*)	Eluant B (% *v*/*v*)
0–3	95	5
3–25	35	65
25–28	30	70
28–33	30	70
33–35	95	5
35–55	95	5

## Data Availability

Not applicable.

## Supplementary Materials

The following supporting information can be downloaded at: https://www.mdpi.com/article/10.3390/molecules28062848/s1: Figure S1: Comparison between the HPLC-MS profiles of native lysozyme HCl (blue) and heat-treated lysozyme HCl (red); Figure S2: Comparison between the mass spectra corresponding to the main peak (labelled as 1 in Figure S1) and the lower one (labelled as 2 in Figure S1) of native lysozyme HCl (on the left, in blue) and heat-treated lysozyme HCl (on the right, in red); Figure S3: Expanded mass spectra corresponding to the main peak (labelled as 1 in Figure S1) and the lower one (labelled as 2 in Figure S1) of native lysozyme HCl (on the left, in blue) and heat-treated lysozyme HCl (on the right, in red); Figure S4: Deconvolution HRMS spectra of native lysozyme HCl and comparison with the theoretical molecular formulas; Figure S5: Deconvolution HRMS spectra of heat-treated lysozyme HCl; Figure S6: Superimposed 1H-13C HSQC spectra recorded in D2O of native lysozyme HCl (red) and heat-treated lysozyme HCl (blue); Figure S7: Superimposed 13C NMR spectra recorded in D2O of native lysozyme HCl (blue) and heat-treated lysozyme HCl (red); Figure S8: Superimposed 1H NMR spectra recorded in H2O/D2O 9:1 of native lysozyme HCl (blue) and heat-treated lysozyme HCl (red); Figure S9: Superimposed 1H-15N HSQC spectra recorded in H2O/D2O 9:1 of native lysozyme HCl (red) and heat-treated lysozyme HCl (blue); Figure S10: Superimposed 13C MAS spectra of native lysozyme HCl (blue) and heat-treated lysozyme HCl (red); Figure S11: Superimposed 13C CP MAS spectra of native lysozyme HCl (blue) and heat-treated lysozyme HCl (red); Figure S12: Graphical representation of Zp values measured at different pH of native lysozyme HCl (on the left) and heat-treated lysozyme HCl (on the right); Figure S13: CD spectra of lysozyme HCl (left) and heat-treated lysozyme (right) in water (0.5 mg/mL); Figure S14: SDS-PAGE analysis.

## Sample Availability

Samples of the compounds utilized in this study are available from the authors.
