# Supplementary material for: Heat-Treated Lysozyme Hydrochloride: A Study on Its Structural Modifications and Anti-SARS-CoV-2 Activity
Source: Molecules. 2023 Mar 21;28(6):2848. doi: 10.3390/molecules28062848 (PMC10054570; doi:10.3390/molecules28062848)
Supplement: Supplementary file 1 [file molecules-28-02848-s001.zip › molecules-2254458-SI.pdf]

Heat-treated Lysozyme Hydrochloride: a study on its structural modifications and anti-SARS-CoV-2 activity

Serena Delbue [1,†], Elena Pariani [2,†], Silvia Parapini [2], Cristina Galli [2], Nicoletta Basilico [1], Sarah D'Alessandro [3], Sara Pellegrino [4], Elena Pini [4], Samuele Ciceri [5], Patrizia Ferraboschi [6] and Paride Grisenti [7,8,*]

[1] Department of Biomedical, Surgical and Dental Sciences, Università degli Studi di Milano, 20133 Milan, Italy
[2] Department of Biomedical Sciences for Health, Università degli Studi di Milano, 20133 Milan, Italy
[3] Department of Pharmacological and Biomedical Sciences, Università degli Studi di Milano, 20133 Milan, Italy sarah.dalessandro@unimi.it
[4] Department of Pharmaceutical Sciences, General and Organic Chemistry Section "Alessandro Marchesini", University of Milan, Via C. Golgi 19, 20133 Milan, Italy
[5] Department of Pharmaceutical Sciences, University of Milan, Via L. Mangiagalli 25, 20133 Milan, Italy
[6] Department of Medical Biotechnology and Translational Medicine, Università degli Studi di Milano, Via Saldini 50, 20133 Milan, Italy
[7] Bioseutica, Landbouwweg 83, 3899 BD Zeewolde, The Netherlands
[8] Bioseutica, Corso Elvezia, 4, 6900 Lugano, Switzerland
* Correspondence: pgrisenti@bioseutica.com
† These authors contributed equally to this work.

INDEX

Figure S1 Comparison between the HPLC-MS profiles of native lysozyme HCl (blue) and heat-treated lysozyme HCl (red).

Figure S2 Comparison between the mass spectra corresponding to the main peak (labelled as 1 in Figure S1) and the lower one (labelled as 2 in Figure S1) of native lysozyme HCl (on the left, in blue) and heat-treated lysozyme HCl (on the right, in red).

Figure S3 Expanded mass spectra corresponding to the main peak (labelled as 1 in Figure S1) and the lower one (labelled as 2 in Figure S1) of native lysozyme HCl (on the left, in blue) and heat-treated lysozyme HCl (on the right, in red).

Figure S4 Deconvolution HRMS spectra of native lysozyme HCl and comparison with the theoretical molecular formulas.

Figure S5 Deconvolution HRMS spectra of heat-treated lysozyme HCl.

Figure S6 Superimposed ^{1}H-^{13}C HSQC spectra recorded in D_2O of native lysozyme HCl (red) and heat-treated lysozyme HCl (blue).

Figure S7 Superimposed ^{13}C NMR spectra recorded in D_2O of native lysozyme HCl (blue) and heat-treated lysozyme HCl (red).

Figure S8 Superimposed ^{1}H NMR spectra recorded in H_2O/D_2O 9:1 of native lysozyme HCl (blue) and heat-treated lysozyme HCl (red).

Figure S9 Superimposed ^{1}H-^{15}N HSQC spectra recorded in H_2O/D_2O 9:1 of native lysozyme HCl (red) and heat-treated lysozyme HCl (blue).

Figure S10 Superimposed ^{13}C MAS spectra of native lysozyme HCl (blue) and heat-treated lysozyme HCl (red).

Figure S11 Superimposed ^{13}C CP MAS spectra of native lysozyme HCl (blue) and heat-treated lysozyme HCl (red).

Figure S12 Graphical representation of Zp values measured at different pH of native lysozyme HCl (on the left) and heat-treated lysozyme HCl (on the right).

Figure S13 CD spectra of lysozyme HCl (left) and heat-treated lysozyme (right) in water (0.5 mg/mL).

Figure S14 SDS-PAGE analyses

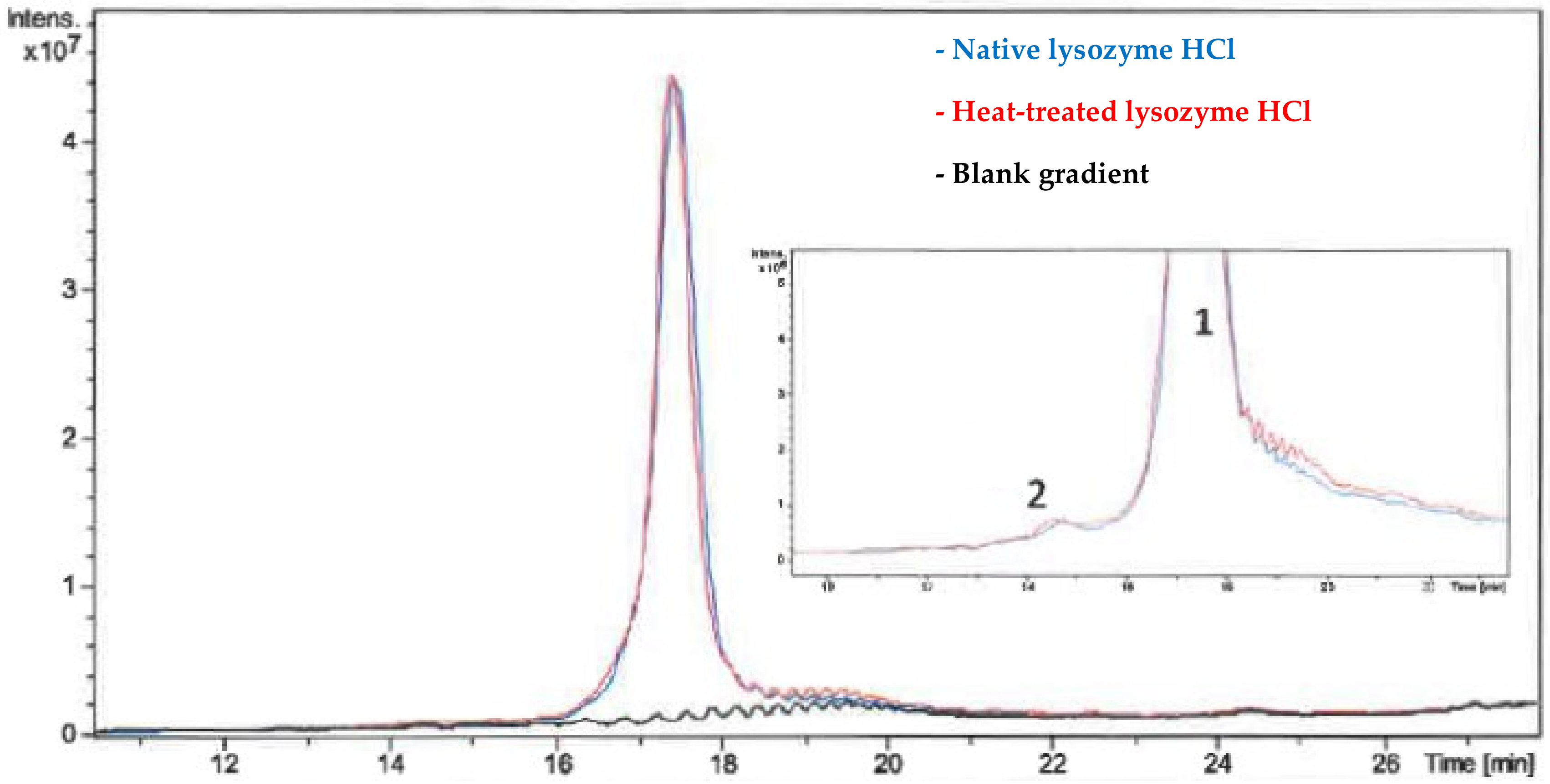

Figure S1. Comparison between the HPLC-MS profiles of native lysozyme HCl (blue) and heat-treated lysozyme HCl (red)

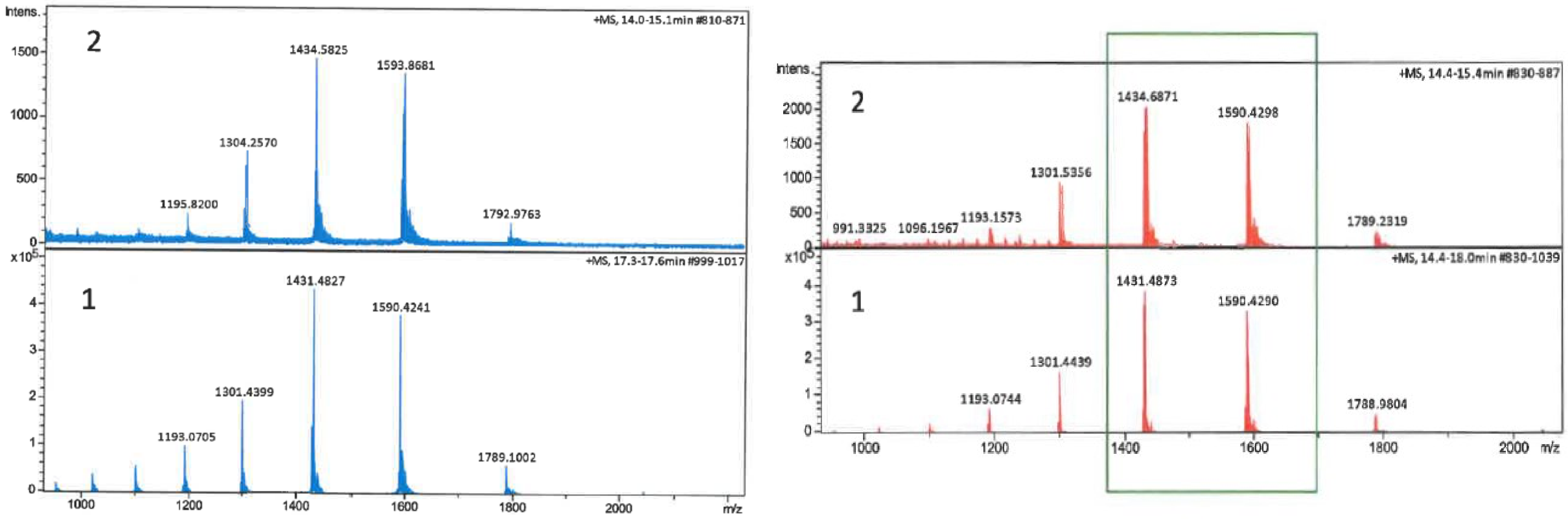

Figure S2. Comparison between the mass spectra corresponding to the main peak (labelled as 1 in Figure S1) and the lower one (labelled as 2 in Figure S1) of native lysozyme HCl (on the left, in blue) and heat-treated lysozyme HCl (on the right, in red).

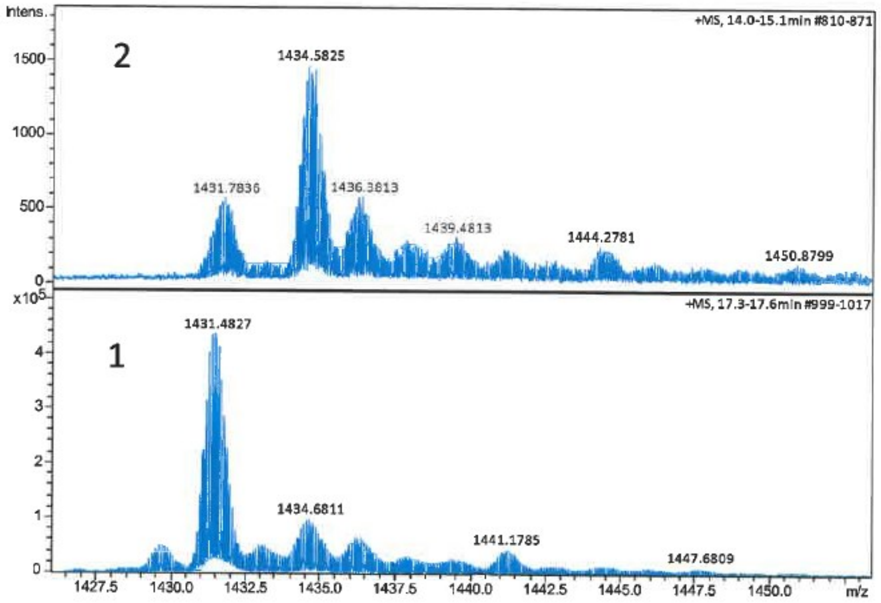

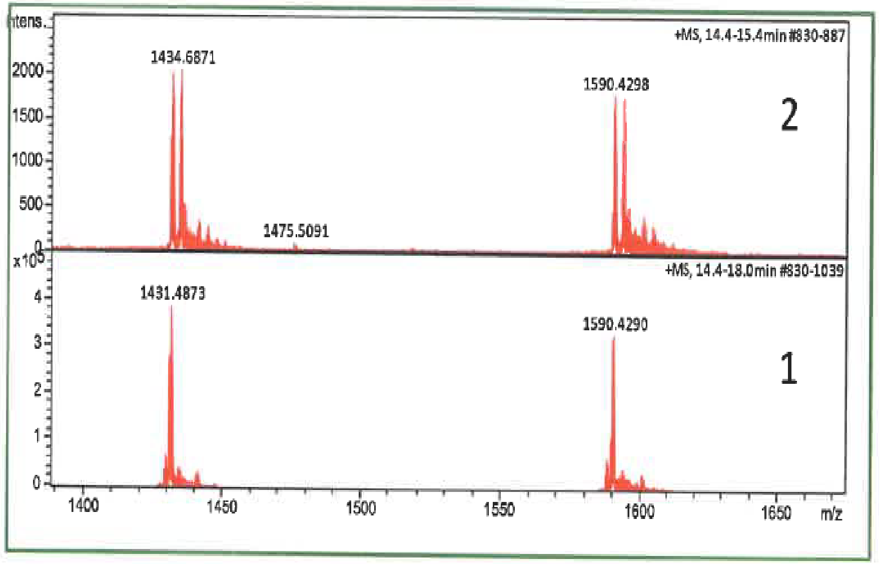

Figure S3. Expanded mass spectra corresponding to the main peak (labelled as 1 in Figure S1) and the lower one (labelled as 2 in Figure S1) of native lysozyme HCl (on the left, in blue) and heat-treated lysozyme HCl (on the right, in red).

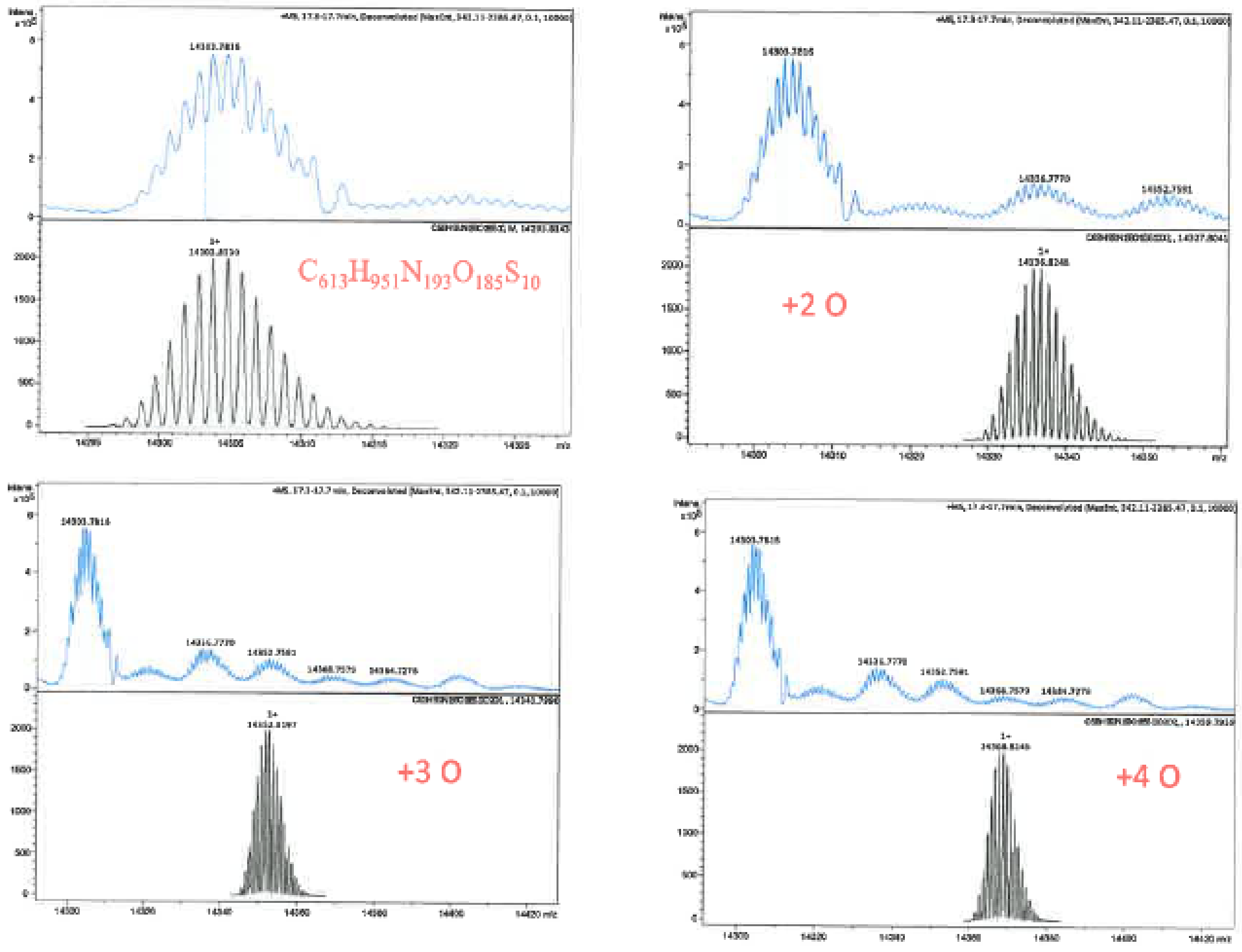

Figure S4. Deconvolution HRMS spectra of native lysozyme HCl and comparison with the theoretical molecular formulas.

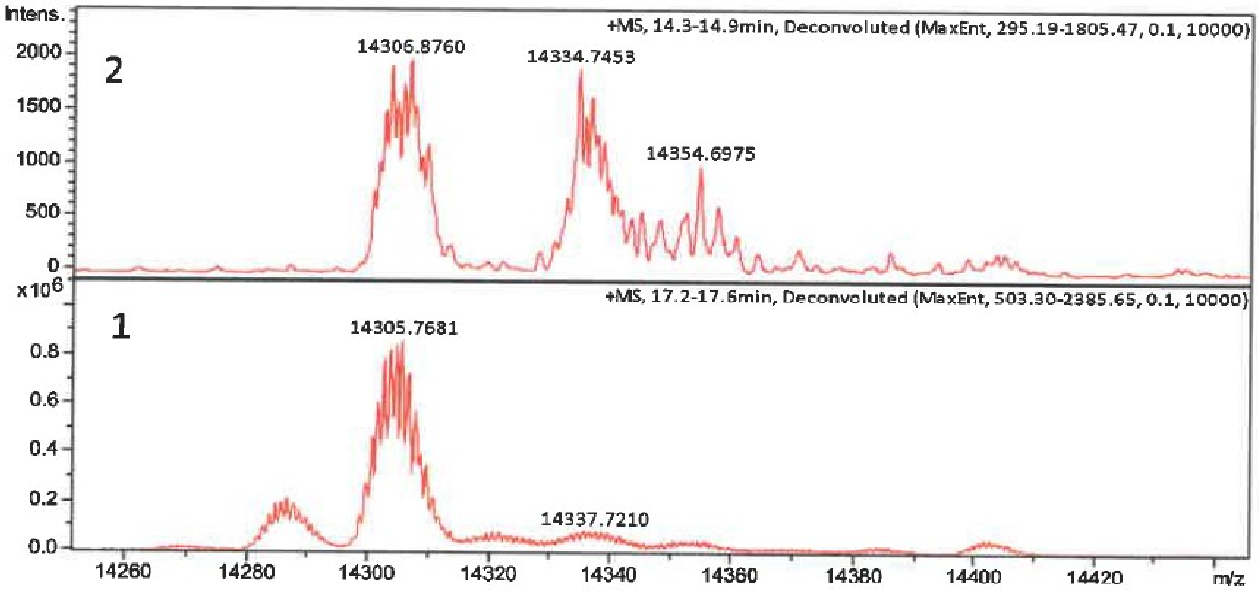

Figure S5. Deconvolution HRMS spectra of heat-treated lysozyme HCl.

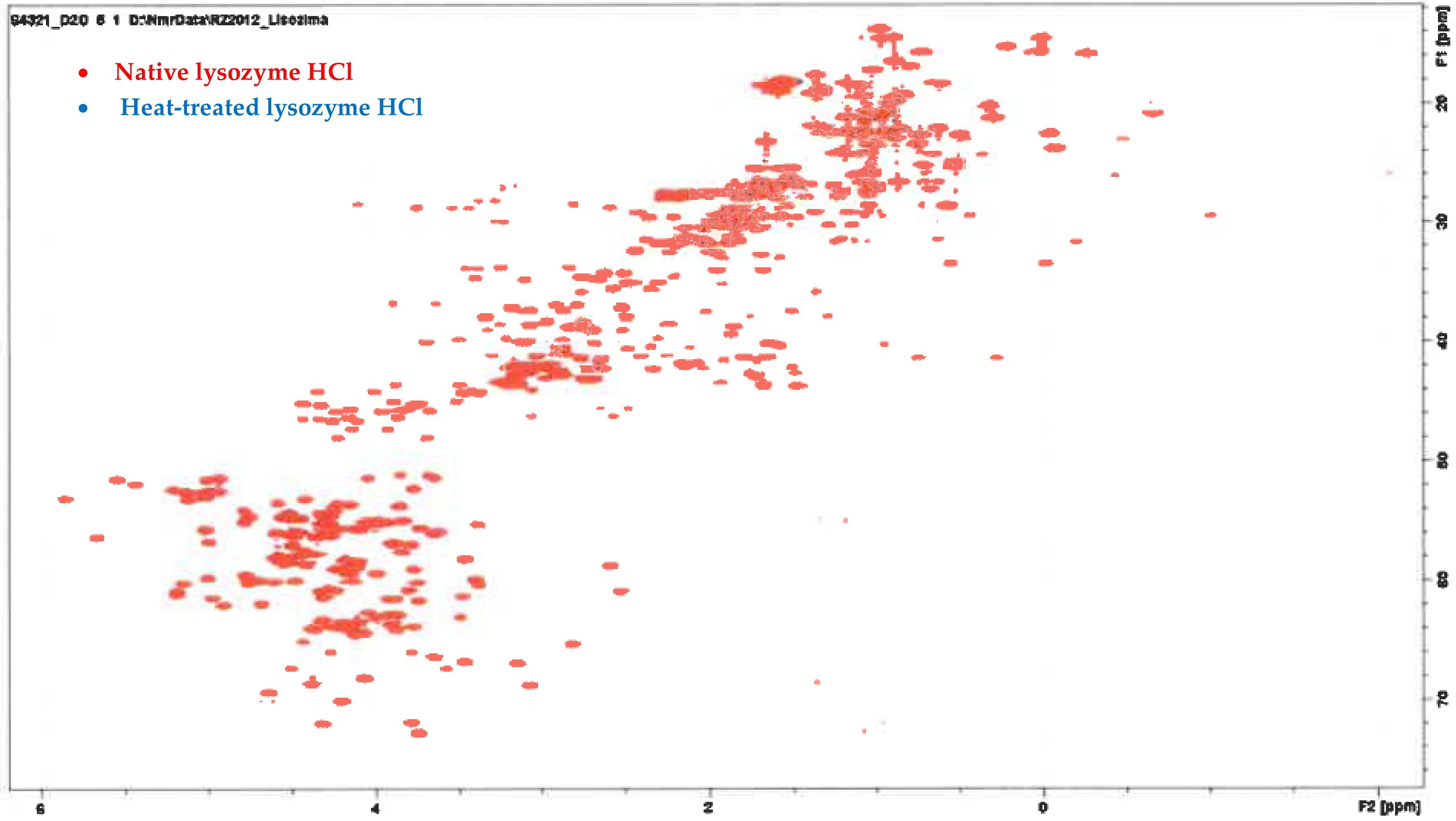

Figure S6. Superimposed ^1H-^{13}C HSQC spectra recorded in D_2O of native lysozyme HCl (red) and heat-treated lysozyme HCl (blue).

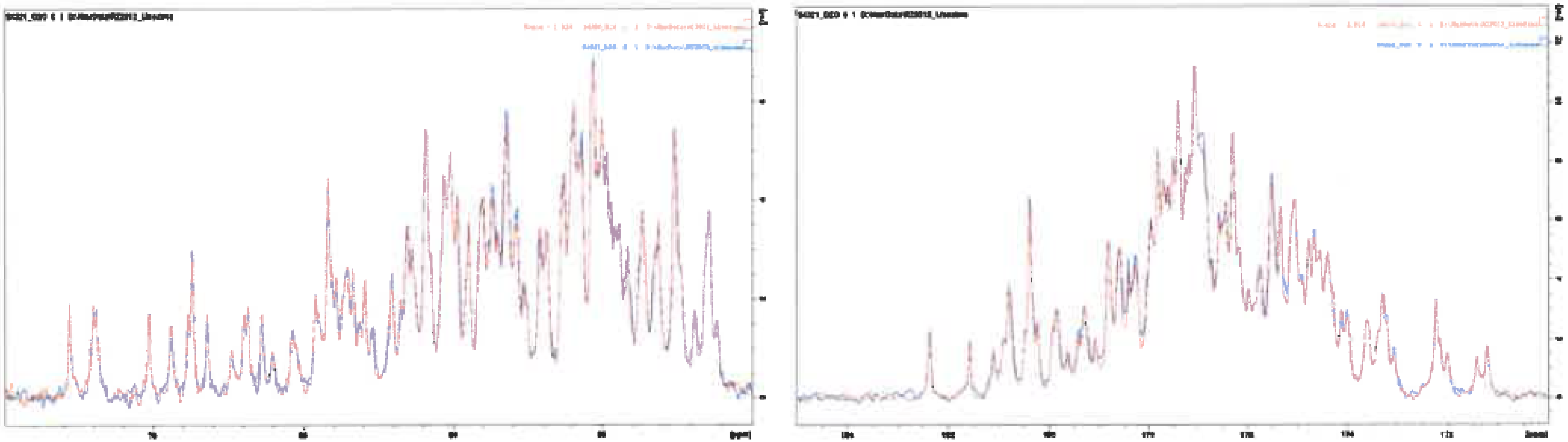

^{13}C-NMR – Zoom on the Cα region.

^{13}C – Zoom on quaternary carbons

Figure S7. Superimposed ^{13}C NMR spectra recorded in D_2O of native lysozyme HCl (blue) and heat-treated lysozyme HCl (red).

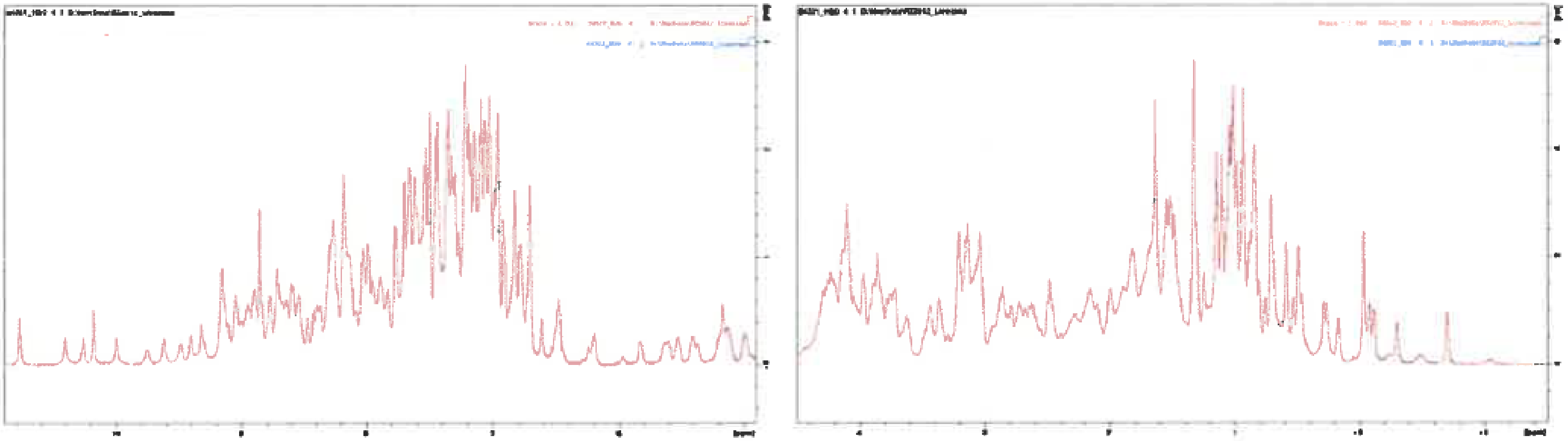

Figure S8. Superimposed ^{1}H NMR spectra recorded in H_2O/D_2O 9:1 of native lysozyme HCl (blue) and heat-treated lysozyme HCl (red).

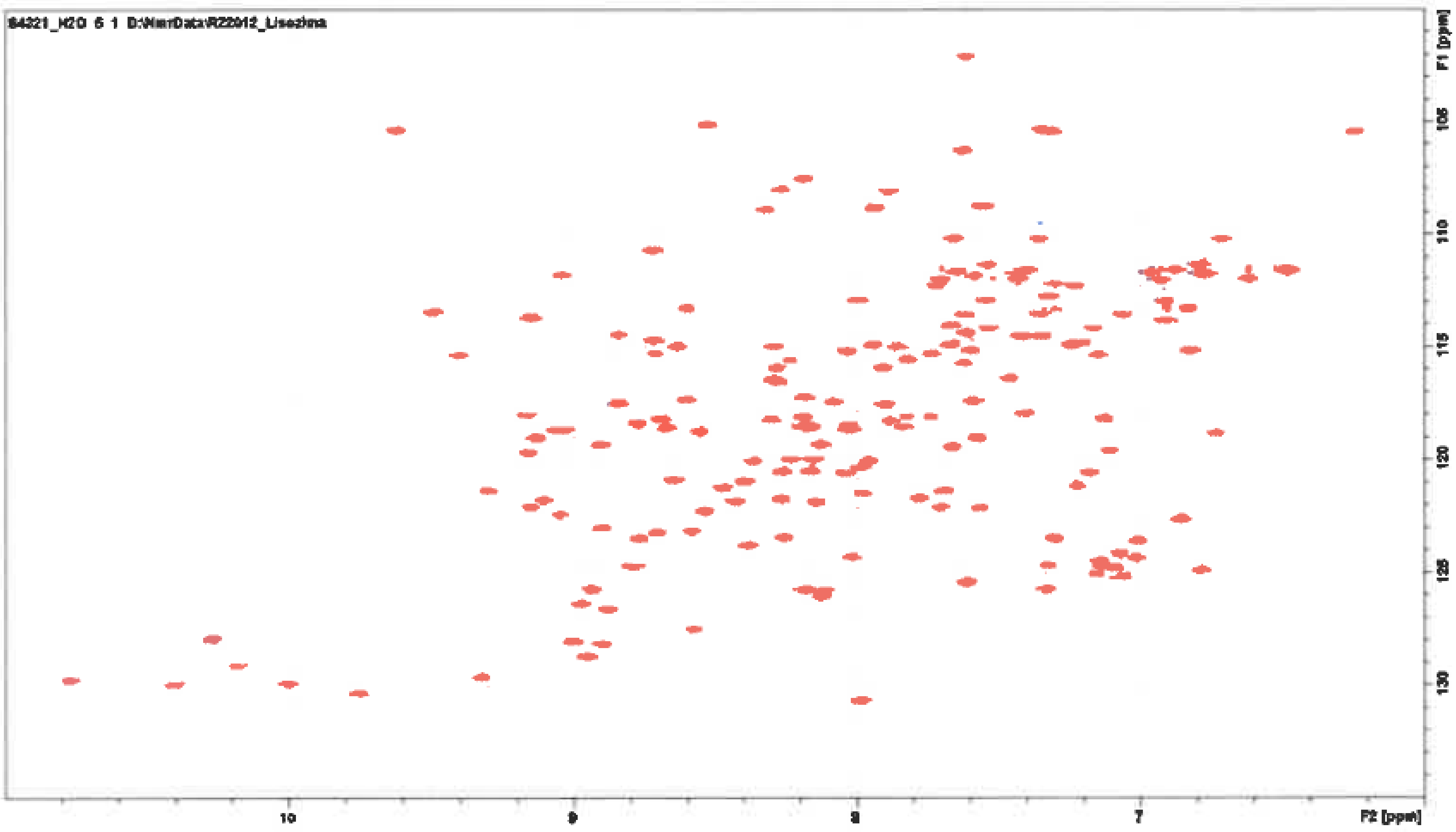

Figure S9. Superimposed ^{1}H-^{15}N HSQC spectra recorded in H_2O/D_2O 9:1 of native lysozyme HCl (red) and heat-treated lysozyme HCl (blue).

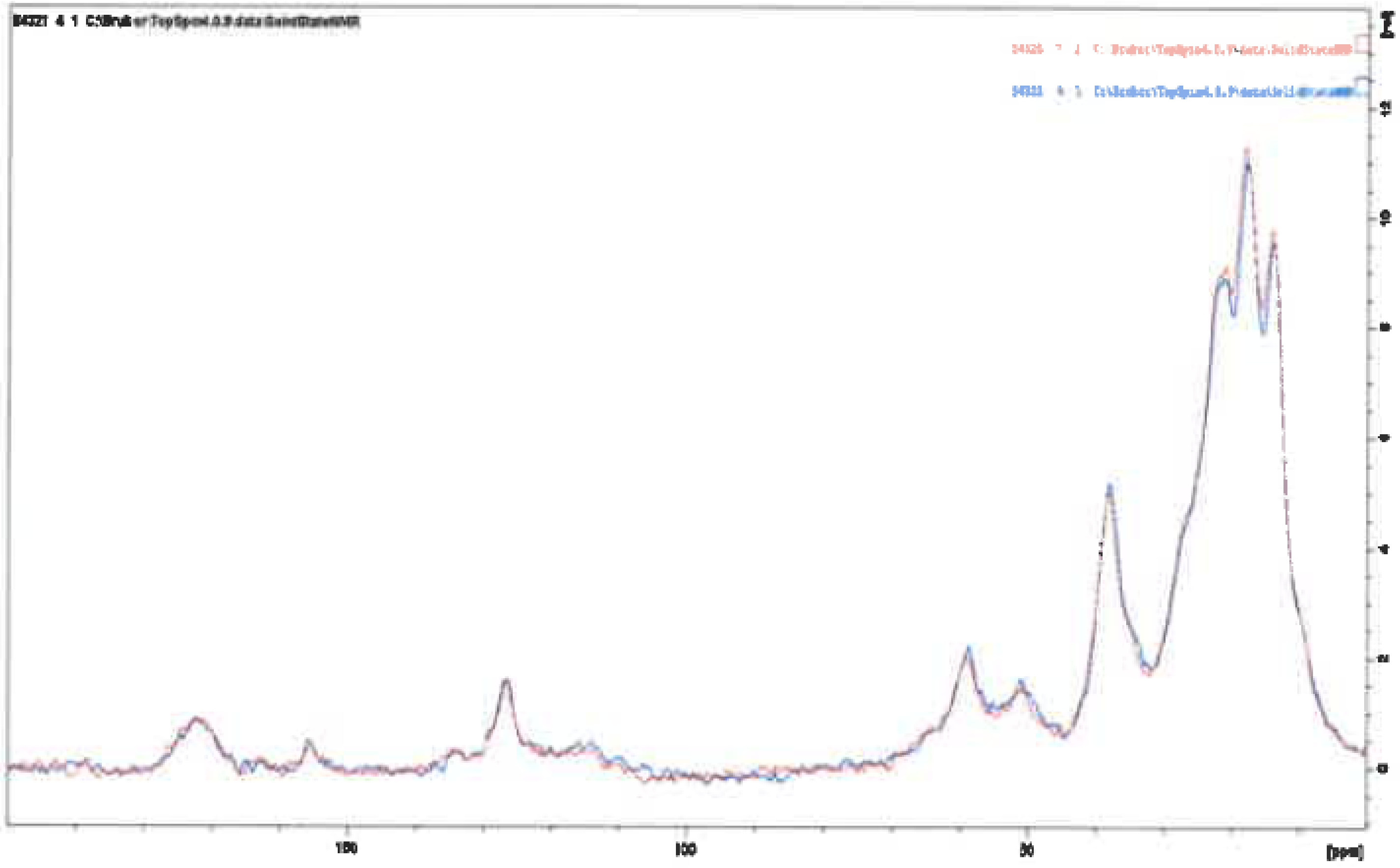

Figure S10. Superimposed ^{13}C MAS spectra of native lysozyme HCl (blue) and heat-treated lysozyme HCl (red).

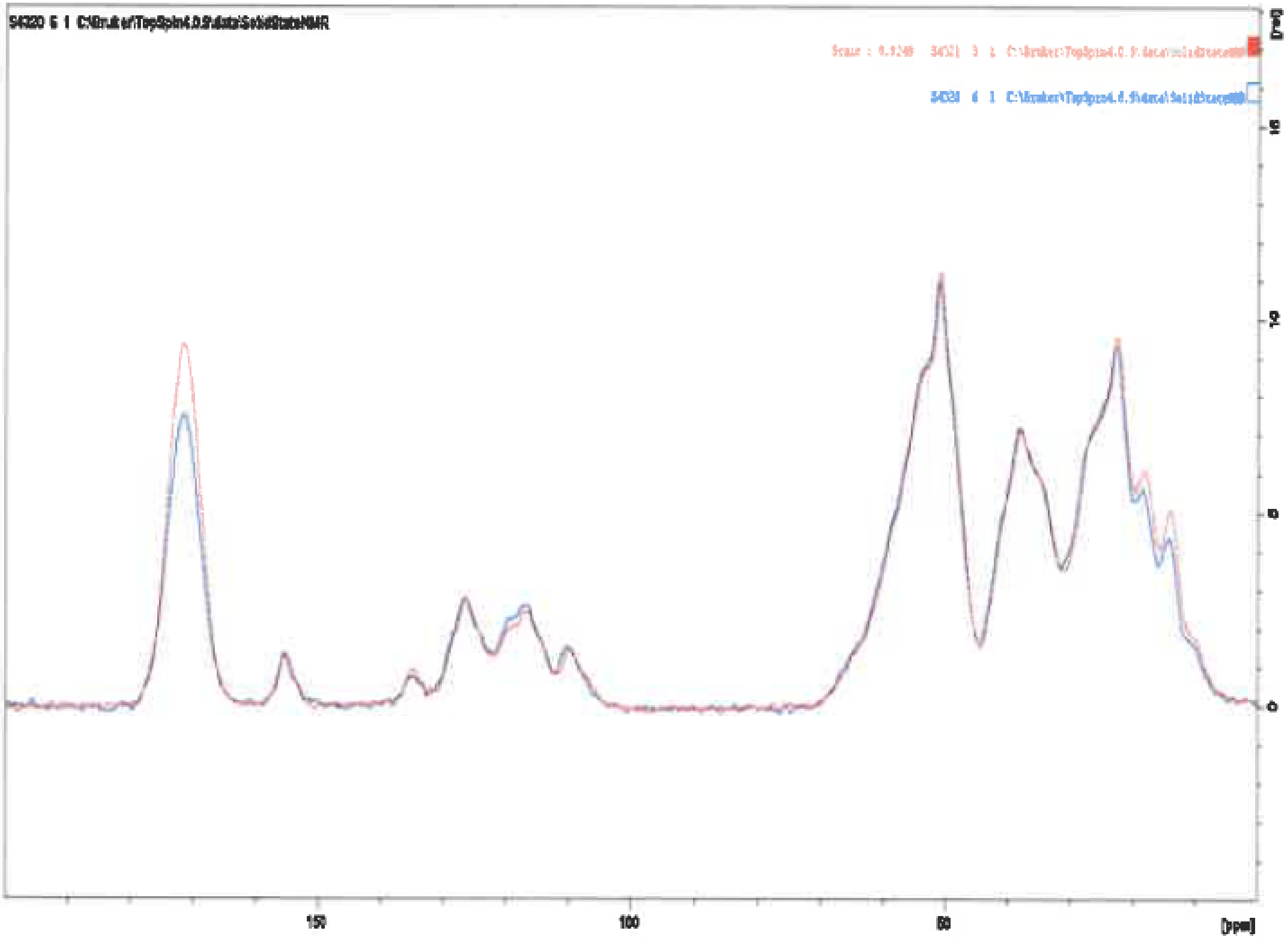

Figure S11. Superimposed ^{13}C CP MAS spectra of native lysozyme HCl (blue) and heat-treated lysozyme HCl (red).

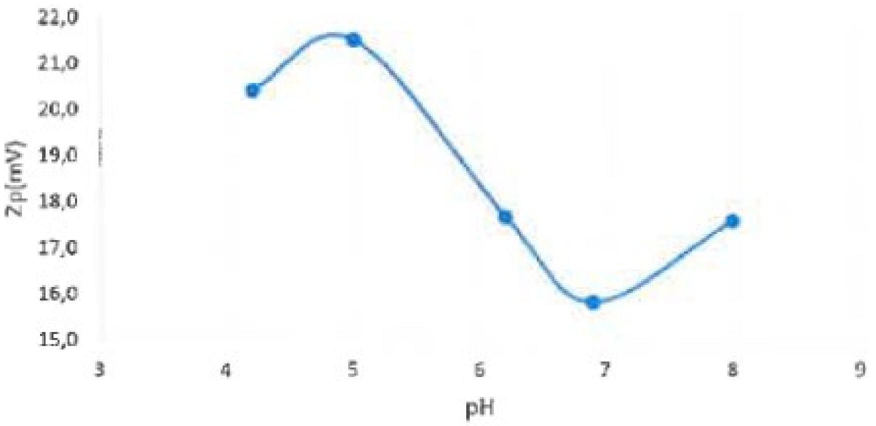

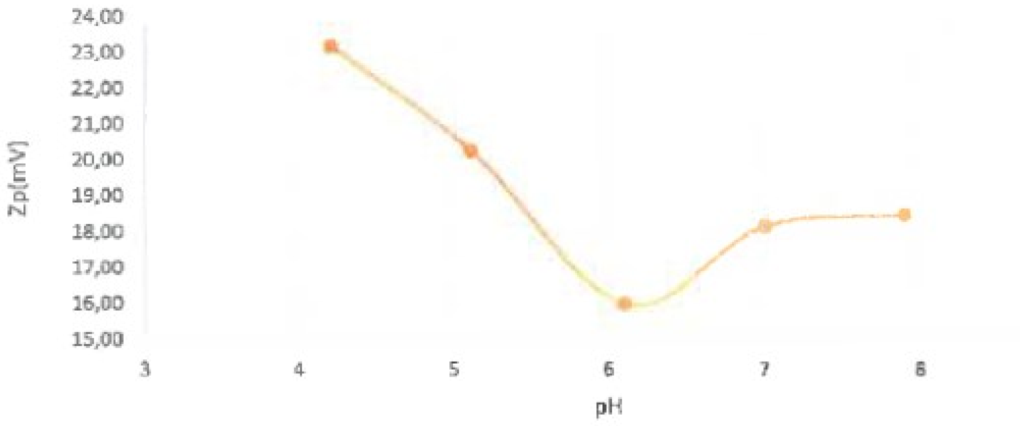

Figure S12. Graphical representation of Zp values measured at different pH of native lysozyme HCl (on the left) and heat-treated lysozyme HCl (on the right).

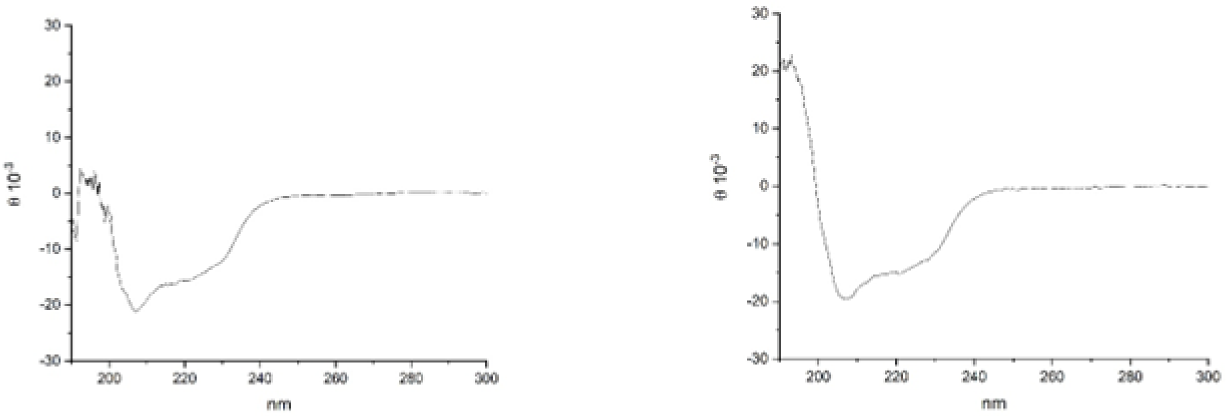

Figure S13. CD spectra of lysozyme HCl (left) and heat-treated lysozyme (right) in water (0.5 mg/mL).

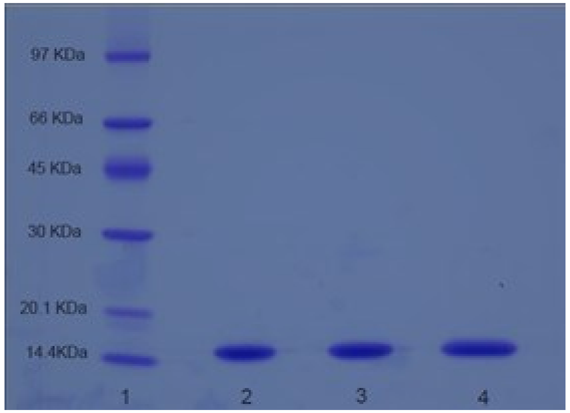

Figure S14. SDS-PAGE analyses.
